# Supplementary material for: Predicting Neoadjuvant Treatment Response in Triple-Negative Breast Cancer Using Machine Learning
Source: Diagnostics (Basel). 2023 Dec 28;14(1):74. doi: 10.3390/diagnostics14010074 (PMC10871101; doi:10.3390/diagnostics14010074)
Supplement: Supplementary file 1 [file diagnostics-14-00074-s001.zip › diagnostics-2726165-supplementary.pdf]

Baseline Characteristics		pCR cases	RD cases
		**n (%)**	**n (%)**
Age			
	≥50	41 (73.2)	24 (64.9)
	<50	15 (26.8)	13 (35.1)
Grade			
	1	3 (5.4)	0 (0)
	2	13 (23.2)	4 (10.8)
	3	40 (71.4)	33 (89.2)
Vital status			
	Alive	33 (58.9)	24 (64.9)
	Dead	23 (41.1)	13 (35.1)
Race			
	AA	31 (55.4)	27 (73)
	EA	21 (37.5)	10 (27)
	missing	4 (7.1)	-

Table S1: Baseline characteristics of study cohort.
AA= African American; EA= European American

Hotspot identification metric	Features considered in hotspots	Image patch accuracy	Patient accuracy	ML algorithm	Parameters
TCs	TCs	0.579	0.579	LDA	Threshold for the significant singular value in the singular value decomposition is 1.42e-05.
	tTILs; sTILs; P; M; TCs	0.566	0.579	LDA	Threshold for the significant singular value in the singular value decomposition is 1.65e-05.
	tTILs; sTILs; P; M	0.576	0.579	SVM	'C': 0.03969968803793212, 'gamma': 0.4847855048642631, 'kernel': 'poly', 'max_iter': -1, 'shrinking': 'false', 'tol': 0.00331983481009374, 'coef0': 0.944961043057087, 'degree': 4
tTILs	tTILs	0.702	0.684	LDA	Threshold for the significant singular value in the singular value decomposition is 0.074.
	tTILs; sTILs; P; M ; TCs	0.722	0.789	LDA	Threshold for the significant singular value in the singular value decomposition is 0.017.
	tTILs; sTILs; P; M	0.739	0.789	LDA	Threshold for the significant singular value in the singular value decomposition is 1.71e-05.
sTILs	sTILs	0.634	0.632	LDA	Threshold for the significant singular value in the singular value decomposition is 3.48e-04.
	tTILs; sTILs; P; M; TCs	0.561	0.684	MLP	'activation': 'relu', 'alpha': 8.404554847932393e-05, 'batch_size': 'auto', 'beta_1': 0.9, 'beta_2': 0.999, 'early_stopping': 'true', 'epsilon': 1e-08, 'hidden_layer_depth': 2, 'learning_rate_init': 0.0024712061205127765, 'n_iter_no_change': 32, 'num_nodes_per_layer': 225, 'shuffle': 'true', 'solver': 'adam', 'tol': 0.0001
	tTILs; sTILs; P; M	0.632	0.632	LDA	Threshold for the significant singular value in the singular value decomposition is 0.011.
sTILs + tTILs	tTILs; sTILs	0.657	0.632	LDA	Threshold for the significant singular value in the singular value decomposition is 2.09e-04.
	tTILs; sTILs; P; M; TCs	0.607	0.737	MLP	'activation': 'tanh', 'alpha': 0.006849763052192261, 'batch_size': 'auto', 'beta_1': 0.9, 'beta_2': 0.999, 'early_stopping': 'true', 'epsilon': 1e-08,

					'hidden_layer_depth': 1, 'learning_rate_init': 0.0002122736659953588, 'n_iter_no_change': 32, 'num_nodes_per_layer': 255, 'shuffle': 'true', 'solver': 'adam', 'tol': 0.0001
	tTILs; sTILs; P; M	0.632	0.632	LDA	Threshold for the significant singular value in the singular value decomposition is 5.27e-03.
**P**	P	0.534	0.526	MLP	'activation': 'relu', 'alpha': 2.9405074940663223e-05, 'batch_size': 'auto', 'beta_1': 0.9, 'beta_2': 0.999, 'early_stopping': 'true', 'epsilon': 1e-08, 'hidden_layer_depth': 3, 'learning_rate_init': 0.0009570549055238582, 'n_iter_no_change': 32, 'num_nodes_per_layer': 137, 'shuffle': 'true', 'solver': 'adam', 'tol': 0.0001
	tTILs; sTILs; P; M; TCs	0.534	0.632	LDA	Threshold for the significant singular value in the singular value decomposition is 4.20e-05.
	tTILs; sTILs; P; M	0.574	0.526	LDA	Threshold for the significant singular value in the singular value decomposition is 8.37e-03.
**M**	M	0.499	0.421	LDA	Threshold for the significant singular value in the singular value decomposition is 0.030.
	tTILs; sTILs; P; M; TCs	0.584	0.579	SVM	'C': 0.5114237001776776, 'gamma': 0.960150807558031, 'kernel': 'rbf', 'max_iter': -1, 'shrinking': 'false', 'tol': 0.00010417453074893361
	tTILs; sTILs; P; M	0.599	0.579	LDA	Threshold for the significant singular value in the singular value decomposition is 4.91e-03.

Table S2: Model parameters.

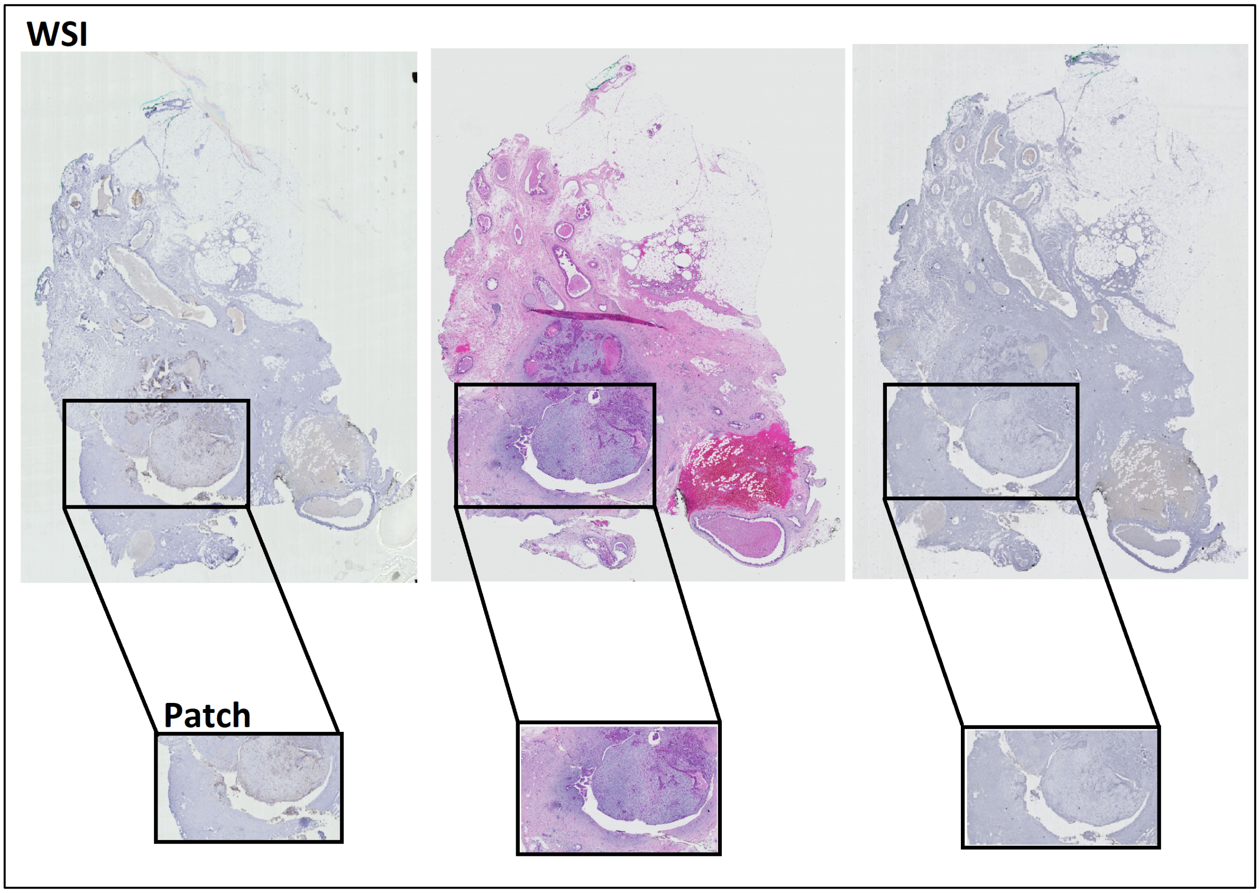

Figure S1. Typical example of WSI triplets of a patient from our dataset. The WSI is the gigapixel image that represents the entire patient tissue biopsy. WSI triplets are registered by 8,000 x 8,000 regions that are further partitioned to 1,000 x 1,000 non-overlapping patches to facilitate the computation.
